# Differential expression of miR-1297, miR-3191-5p, miR-4435, and miR-4465 in malignant and benign breast tumors

**DOI:** 10.22038/ijbms.2020.44581.10421

**Published:** 2020-08

**Authors:** Abbas Mosapour, Fatemeh Soghra Karami Tehrani, Morteza Atri

**Affiliations:** 1 Cancer Research Lab, Department of Clinical Biochemistry, Faculty of Medical Sciences, Tarbiat Modares University, Tehran, Iran; 2 Department of Surgery, Faculty of Medicine, Tehran University of Medical Sciences, Tehran, Iran

**Keywords:** Biomarker, Breast cancer, Diagnosis, miR (MicroRNA), OncomiR

## Abstract

**Objective(s):**

MicroRNAs (miRs) are a class of small non-coding RNAs which are associated with tumor growth and progression. In the present study, we assessed the expression of selected miRs in malignant, benign, and adjacent normal breast tissues.

**Materials and Methods:**

The expression of miR-1297, miR-3191-5P, miR-4435, and miR-4465 were evaluated in malignant (n=50), benign (n=35), and adjacent normal breast tissues (n=20) using qRT-PCR. Receiver operating characteristic (ROC) curves and the area under the ROC curve (AUC) were generated for evaluating the diagnostic values of miRs. To evaluate diagnostic efficacy, miRs-based score was obtained using the logistic regression model.

**Results:**

Among malignant tumors, the expression of miR-1297, miR-3191-5p, and miR-4435 was significantly lower (*P*=0.024, *P*<0.001 and *P*=0.031), respectively. The expression of miR-4465 was higher (*P*=0.023) than that of normal tissue. The expression of these miRs was lower than those of benign tumors (*P*<0.01, *P*<0.001, *P*<0.0001, and *P*<0.01, respectively). We observed a positive correlation between miR-4465 expression levels and tumor stage (*P*=0.042) and a negative correlation with grade and Ki-67 score (*P*<0.05). The AUCs for miR-1297, miR-3191-5p, miR-4435, and miR-4465 in malignant tumors versus normal tissues were 0.784, 0.700, 0.976, and 0.865 and versus benign tumors they were 0.938, 0.857, 0.981, and 0.785, respectively. The optimal logit(P) value of 0.262 distinguished malignant from normal subjects with a sensitivity of 0.91, specificity of 0.85, and an overall accuracy of 0.89.

**Conclusion:**

The panel of these miRs are suggested as possible onco-miRs(miR-4465) or tumor suppressor-miRs (miR-3191-5P, miR-1297, miR-4435). Overall, our results indicated that these miRs could be introduced as diagnostic biomarkers in breast cancer patients.

## Introduction

Breast cancer is known as the second most common type of cancer as well as the main reason for death from cancer among women (1-3). Finding a non-invasive biomarker for detecting breast cancer in early stages is a very important challenge in the diagnosis and management of this disease. 

The abnormal miRs expression levels can be associated with various types of cancer (4, 5). It has been shown that miRs are able to regulate several cellular and cancer-related mechanisms including cell-cycle control, metabolism, cell proliferation, apoptosis, invasion, and metastasis (6-8). Tissue-specific and circulating miRs have been recognized as some of major regulatory factors in signal transduction and other biological pathways (9-11). miRs as new laboratory biomarkers and therapeutic targets have been recommended (12, 13). An miR-based biomarker can be used, either as a single biomarker or a biomarker panel. For instance, miR-21 could be used as a single biomarker for chemoresistance in esophageal squamous cell carcinoma (14). However, it can also be used together with another miR (miR-375) and prostate-specific antigen (PSA) (a non-miR indicator) as a biomarker panel for early diagnosis of prostate cancer (15, 16). 

It has been found that miR-1297 plays a key role in hepatocellular carcinoma and colorectal and prostate cancer cells, since it suppresses the growth, migration, and invasion of tumor and induces cell apoptosis (17). In addition, miR-1297 together with miR-4465, suppresses non-small cell lung cancer (NSCLC) proliferation (18). However, in testicular germ cells, laryngeal squamous cell carcinoma, and cervical carcinoma, miR-1297 facilitates the expression of PTEN and contributes to cell invasion (19, 20).

A panel of nine miRs (miR-15a, miR-18a, miR-107, miR-133a, miR-139-5p, miR-143, miR-145, miR-365, and miR-425) has been introduced for discriminating breast cancer patients from normal subjects (21). The serum levels of miR-484 were higher in patients with breast cancer (22). In an investigation performed among the Japanese population, an assay of serum miR was suggested as biomarker (microarray-based) for early diagnosis of breast cancer (23). A panel of five miRs (miR-1246, miR-1307-3p, miR-4634, miR-6861-5p, and miR-6875-5p) was also introduced as a biomarker for early detection of breast cancer with 89.7% accuracy (24). Different cellular and extracellular miR profiles have been shown in breast cancer and its cell lines as it was observed that serum levels of miRs do not reflect their levels of expression in the malignant cells (25). Moreover, several studies have shown that the expression levels of specific serum miRs have not indicated to be similar to those of tumor tissues. Of 19 miRs up-regulated in breast cancer tissues, only 2 miRs were also up-regulated in serum (26). 

Considering the predictions of bioinformatics (web-based online programs), the data of previous reports and comprehensive review of relevant literature (13, 18, 21-23, 25, 27-34), the aim of this study was to identify some miRs including miR-1297, miR-3191-5P, miR-4435, and miR-4465 in benign and malignant breast tumors. Moreover, we assessed the correlation of these miRs with clinicopathological features. 

## Materials and Methods

***Patients and tissue sampling***

The present research was done based on the Declaration of Helsinki (local ethical approval), and written informed consents were received. 50 subjects with breast ductal cell carcinoma and 35 breast tumors of fibroadenoma (benign) referring to Day and Bahman Hospitals, Tehran, Iran, were included. Through surgery, their tumor tissues were sampled between October 2015 and July 2017. All patients diagnosed with primary breast cancer and no therapy prior to surgery, were included, regardless of their age, race, or nationality. There are no restrictions on tumor stage, grade, tumor size, and lymph node involvement. Tumor specimens from non-necrotic proliferative regions and normal tissues (35) were taken away (a pathologist confirmed the histopathology of all specimens as soon as they were collected). Patients were excluded if they had multifocal cancer / prior malignancy or any kind of therapy (e.g. adjuvant treatment). Immediately, fresh tissues were carefully clipped of adipose and necrotic tissues and were stored at −80 ^°^C. The results were evaluated based on age, menopausal status, estrogen receptor (ER), progestrone receptor (PR), Human Epidermal growth factor Receptor 2 (HER2), grade, stage, Ki67% score, and size of tumors. Immunohistochemistry was applied to assess prognostic biomarkers, mentioned in pathology reports. Demographic characteristic of studied participants has been shown (Table 1). 

***RNA Isolation, cDNA Synthesis***

The Mikro-Dismembrator device (Braun, Germany) was used for obtaining pulverized fine powder of frozen tissues. For the extraction of total RNA that contained miRs, RiboEx reagent (recommended by manufacturer) was applied to the obtained powder (Cat no. 301-001; GeneAll, South Korea) and the extracted RNA was stored at −80 ^°^C. The concentration of RNA was measured by a UV spectrophotometer at 260 nm. A260/A280 ratio on Nanodrop 2000 UV-Vis spectrophotometer (no. ND2000; Waltham, MA) was used to determine the purity and integrity. To remove possible contamination of DNA, treatment of extraceted RNA was done using DNase (Thermo fisher, USA). Then reverse transcription into first-strand cDNA was performed by Revert M-MuLV reverse transcriptase (Fermentas) using random hexamer (RH) and Oligo dT primers in a reaction volume (20 µl) as recommended by the manufacturer (Table 2). In brief, to generate cDNA of miR-1297, miR-14435, miR-3191-5p, miR-4465, and miR-16-5p; 1 μg of RNA, 1 μl (50 nM) stem-loop RT primer, and x μl DEPC-treated water (to final volume 13.4 μl) were first mixed gently and centrifuged briefly. Then they were incubated at 70 ^°^C for 5 min before quenching on ice. After that, 1 mM of each of the four deoxynucleotide triphosphates (dNTPs[10 mM each]), 0.5 μl ribonuclease inhibitor (RNasin [40 U/μl]), 1 μl M-MLV reverse transcriptase (200 U/µl), and 4 μl 5x first-strand buffer (MBI Fermentas, USA) were added together to make up a final volume of 20 μl reaction mix. The reaction mix was incubated in a Gradient Thermal Cycler (Bio-Rad Laboratories, Inc., California, USA) for 30 min at 16 ^°^C, and 60 min at 42 ^°^C. The reverse transcriptase was inactivated at 70 ^°^C for 5 min and then stored at -20 ^°^C. 

***Quantitative Real-time PCR***

We employed qRT-PCR for assessing miR-1297, miR-3191-5P, miR-4435, and miR-4465 cDNA using primers (LIGO Macrogen) that are specifically designed for miRs (Table 3). We also used Master Mix (RealQ Plus 2x master mix green without ROX SYBR® Green) in a Real-Time PCR system (Rotorgene Q Real-Time System; Qiagene co.) according to instructions. Identical PCR was performed using 2 μl of cDNA. The relative expression levels of miRs were normalized to U6 and miR-16 as housekeeping genes. The reaction was started at 50 ^°^C for 2 min and at 95 ^°^C for 15 min, then 40 cycles at 95 ^°^C for 15 sec and 60 ^°^C for 30 sec. The threshold cycle (Cq/Ct) results were determined by Rotorgene Q system software (Qiagene co), with default settings. The mean Ct of the duplicate analysis of each sample was considered. To determine relative gene expression level,2-∆∆cq analysis was used (37). These data were expressed as median fold changes and were analyzed using the GenEx software version 2.5 (MultiD Analyses AB, Sweden) and REST software.

***Statistical analysis***

The results were presented as relative fold change (RFC) in three independent studies and the differences were analyzed by Spearman and Mann-Whitney U tests in which the probability values less than 5% (*P*<0.05) were considered significant and it was indicated by an asterisk in the figures. Data were analyzed using GraphPad Prism statistical software 6(CA, USA) and SPSS Statistics 20. The sensitivity and specificity of miR expression were evaluated based on ROC curves and AUC (38). Additionally, binary logistic regression analysis was also used.

## Results

***Characteristics of participants***

Clinicopathological characteristics of malignant, normal, and benign breast tissues have been shown in Table 1. At the time of tissue collections, the median ages of patients were 50.49 ±11.48 years for malignant and 35.62±13.76 years for benign tumors. In addition, tumor biology was distributed as c-erbB2^+^ in 38.0%, c-erbB2^-^ in 62%, ER/PR^+^ in 62.6%, and ER/PR^-^ in 37.4% of cases. 

***Expression of miRs***

**Figure 1:**
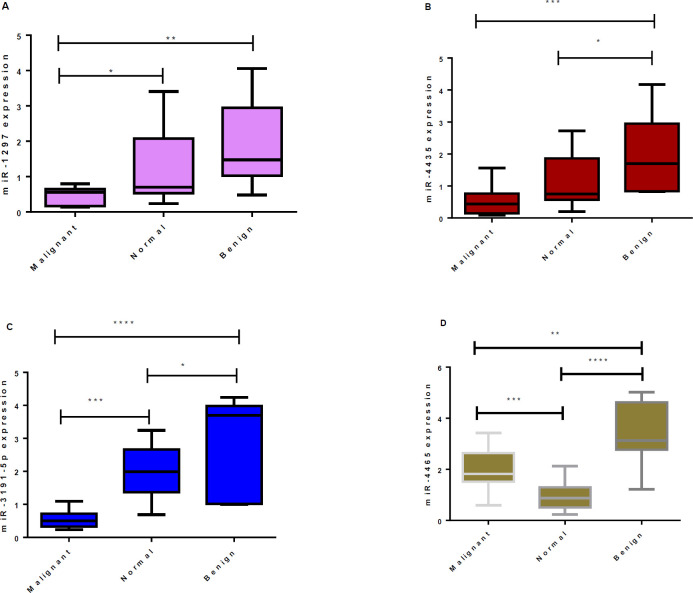
Tukey box plots of miR-1297 (A), miR-4435 (B), miR-3191-5P (C), and miR-4465 (D) levels in malignant, normal, and benign breast tissue samples. qPCR results (relative fold change) data was used for these plots. One-way ANOVA and Tukey-Kramer post-tests were used to identify significant differences. *P<0.05, **P<0.01, and ***P<0.001

The relative expression levels of miR-1297 in the malignant tumors were significantly lower compared with normal as well as benign tissues (*P*<0.05 and *P*<0. 01, respectively; Figure 1A). The relative expressions of miR-4435 in malignant breast tumors were indicated to be significantly lower in comparison with benign tumors (*P*<0.01). Moreover, regarding miR-4435, it was higher in benign tumors compared with normal tissues; however, it was not statistically significant (*P*>0.05; Figure 1B). Considering malignant tumors, the relative expression of miR-3191-5p was significantly lower in comparison with the normal tissues as well as benign tumors (*P*<0.001 and *P*<0.0001, respectively)(Figure 1C). However, in malignant tumors, the miR-4465 expression was significantly higher than that of normal tissues (*P*<0.001) and lower than that of benign tumors (*P*<0.01, Figure 1D). Regarding the expression levels of miR-1297 between benign and normal tissue, we did not find significant changes (*P*>0.05); however, regarding benign tumors, relative expression of miR-4435, miR-31915p, and miR-4465 was higher than that of normal tissue (*P*<0.05, *P*<0.05, and *P*<0.001, respectively).

***Diagnostic value of miRs***

**Figure 2:**
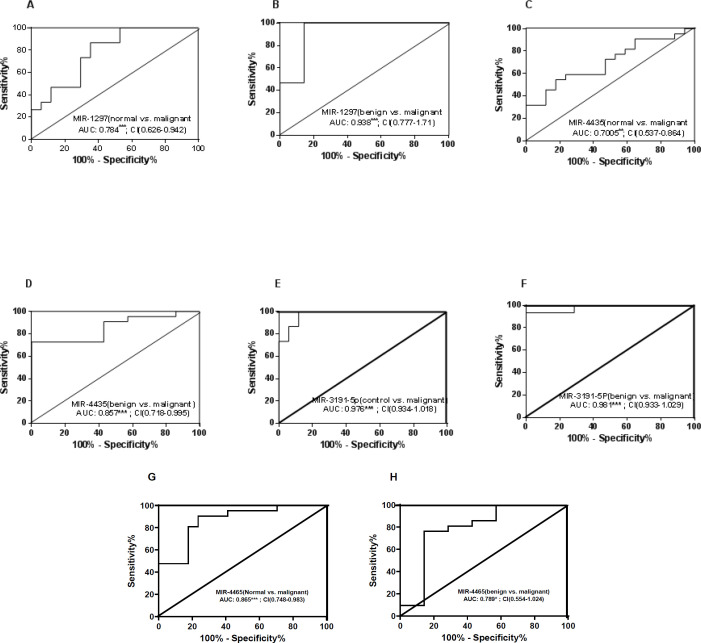
ROC curves of miR-1297 (normal vs malignant) (A), miR-1297 (benign vs malignant) (B), miR-4435 (normal vs malignant) (C), and miR-4435 (benign vs malignant) (D), miR-3191-5P (normal vs malignant) (E), and miR-3191-5P (benign vs malignant) (F), miR-4465(normal vs malignant) (G), and miR-4465 (benign vs malignant) (H). qPCR data of miR expression was used for generating ROC curves in tissue samples of normal & benign versus malignant. AUC and 95% Confidense Intervals (CI) are shown. **P<0.01, ***P<0.001

There was a 0.660-fold down-regulation for miR-1297 (*P*<0.05), a 0.788-fold for miR-4435 (*P*<0.05), and a 0.535-fold for miR-3191-5p (*P*<0.001) in the malignant tumors compared with that of controls, whereas in the malignant tumors, the miR-4465 expression level was higher than that of controls (0.976 fold) (*P*<0.01, Figure 1). To discriminate normal from malignant tissues, AUC for miR-1297, miR-4435, miR-3191-5P, and miR-4465 was found to be 0.784 (95% CI, 0.626-0.942; *P*<0.001), 0.7005 (95% CI, 0.534- 0.864; *P*<0.01), 0.976(95% CI, 0.934- 1.018; *P*<0.001), and 0.865(95% CI, 0.748- 0.983; *P*<0.001); respectively. Moreover, for discriminating benign from malignant, AUC for miR-1297, miR-4435, miR-3191-5P, and miR-4465 was found to be 0.938(95% CI, 0.777-1.71;* P*<0.001), 0.857(95% CI, 0.718- 0.995; *P*<0.001), 0.981 (95% CI, 0.931-1.029; *P*<0.001), and 0.789 (95% CI, 0.554-1.024; *P*<0.05), respectively (Figure 2). As a whole, the results propose that these miRs could be applied for discriminating malignant from normal tissues with a sensitivity of 73.3, 59.09, 86.67, and 80.95 and a specificity of 70.59, 76.47, 94.12, and 82.35 for miR-1297, miR-4435, miR-3191-5p, and miR-4465, respectively; and also to differentiate malignant from benign tumors with a sensitivity of 93.33, 72.73, 93.33, and 66.19; and detection specificity of 85.71, 84.73, 92.69, and 76.71 for mentioned miRs, respectively (Figures 2 A-H).

***Logistic regression analysis***

**Figure 3:**
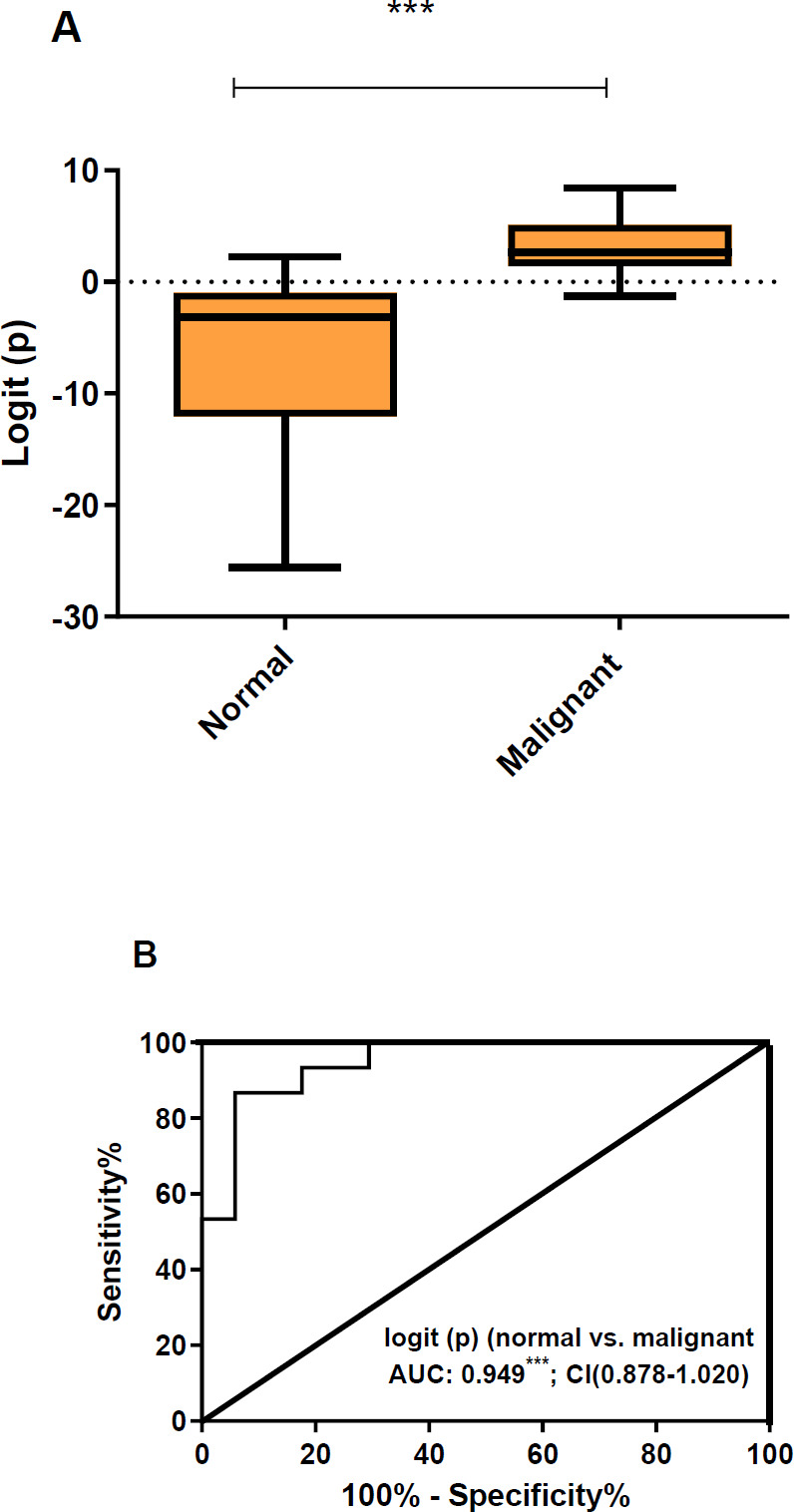
Values of logit(P) in normal and malignant tissue samples. A: values of logit(P)(box plots) shown in normal and malignant samples. Student’s unpaired t-test was used for determining significant differences. B:ROC curve of the logit(P) value in normal vs malignant. AUC and 95% confidence intervals (CI) is shown. ***P<0.001

QRT-PCR was performed for the analysis of expression levels of miR-1297, miR-4435, miR-3191-5p, and miR-4465 in malignant and normal tissues and the data were used for binary logistic regression. The mean logit(P) value of malignant tumors, 2.96 (95% CI[ 1.510- 4.346]), was found to be significantly higher than that of nomal tissues, -7.281 (95% CI[- 11.37 – (- 3.636]) (*P*<0.001) (Figure 3A). AUC was 0.949 (95% CI, 0.878 -1.020; *P*<0.001) (Figure 3B), which indicated the elevated accuracy and discriminated normal tissue from malignant. A logit(P) of 0.265 was used for optimal cutoff (Figure 3B) by which normal and malignant samples were identified (Table 4). In addition, the established miR classifier assigned correctly 38 out of 43 tested cases, proposing a sensitivity of 0.91 (95% CI, 0.82-1.00), specificity of 0.85 (95% CI, 0.71-1.00), and accuracy of 0.89 (95% CI, 0.80-1.00).

***Correlation with clinicopathological features***

**Figure 4:**
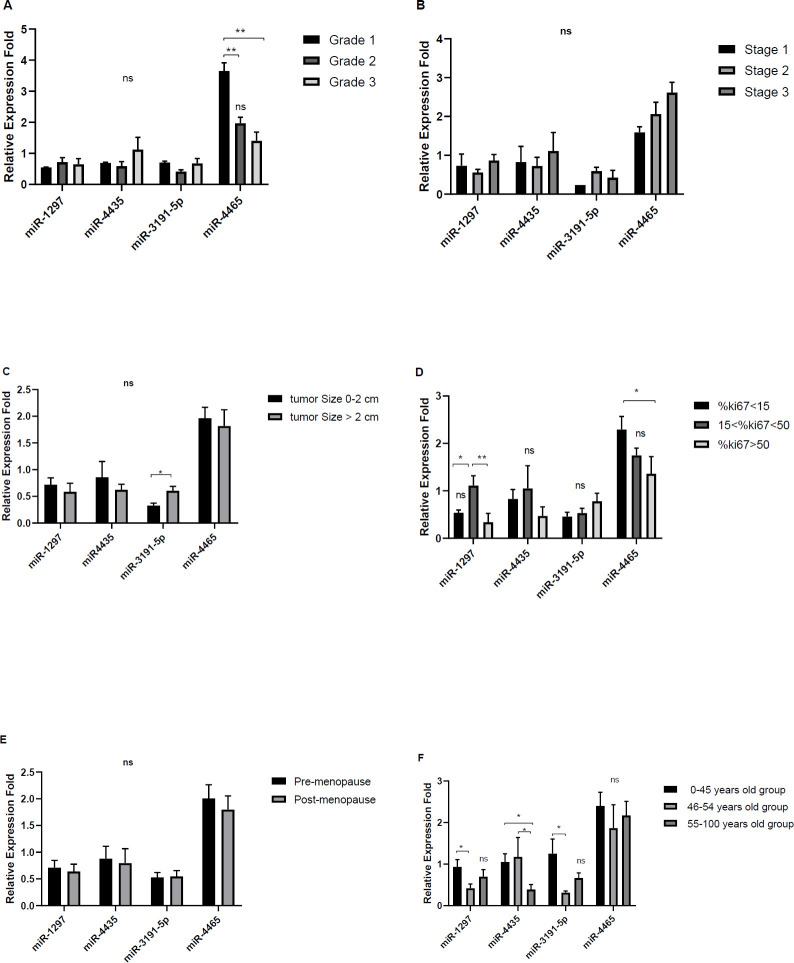
Relative expression levels of miRs in patients according to tumor Grade (A), tumor Stage (B), tumor size (C), %KI 67 grouping (D), menopause status (E), and patient age (F). (*P<0.05; **P<0.01; ns not significant)

**Figure 5:**
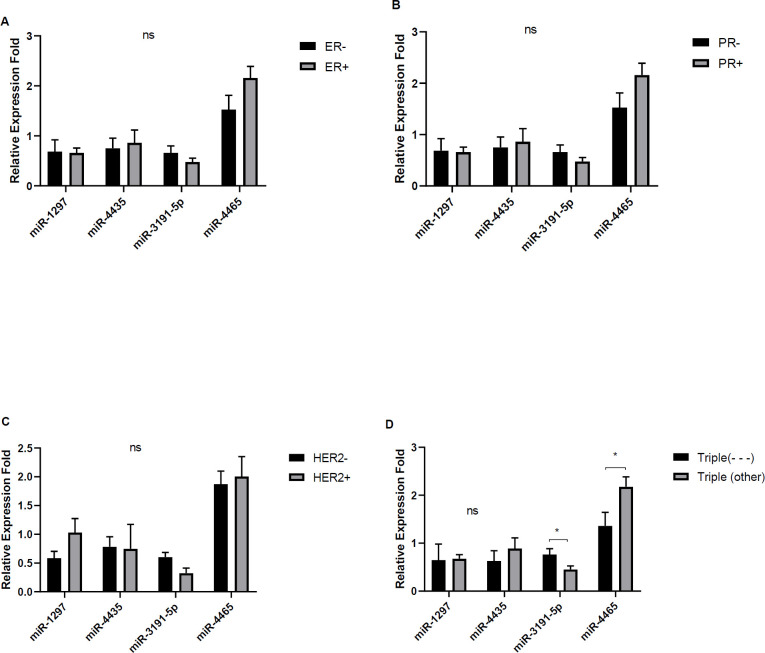
Relative expression levels of miRs in patients according to status of estrogen receptor(ER) (A), status of progesterone receptor (36) (B), HER2 (C), and triple status (D). (*P<0.05; ns not significant)

The relative expression levels of miR-4465 were significantly higher in grade I tumors compared with grades II and III (Figure 4A, *P*<0.01). No significant correlation was observed between the expression of miR-1297, miR-4435, and miR-3191-5p and the grades. The expression levels of miR-4465 were positively associated with tumor stage (*P*<0.05). The expression levels of miR-3191-5P and miR-4465 were higher and lower among triple negative malignant tumors, respectively (Figure 5D, *P*<0.05). However, we found no significant difference between the expression of miRs and ER, PR, and HER2 status, although a slight reduction was found for miR-4465 expression in ER- PR- HER2- tumors (*P*>0.05). It was shown that the expression of miR-3191-5p directly (*P*>0.05) and expression of miR-4465 inversely correlated with the Ki-67% scores (*P*<0.05). In addition, miR-4465 and miR-1297 expression levels were higher in the tumors of ki-67%<15 when compared with those of ki-67%>15 (*P*<0.05).

Regarding the relationship with patient’s age, it was revealed that the expressions of miR-3191-5p and miR-1297 were higher in <45 years old patients than in > 45 years olds (*P*<0.05), whereas miR-4435 expressions were higher in 46-54 year olds than in others (Figure 4F, *P*<0.05). There were positive correlations between miR-4465 expression and ER+ or PR+ or HER2+ (*P* >0.05) while showing a negative correlation with ER-PR-HER2- (*P*<0.05). There were no significant differences between other miRs and ER, PR and HER2 status, stages, status of menopause, and tumors size (Figures 5 A-D).

**Table1:** Clinicopathological characteristics of study populations

	Malignant	Benign	Normal
Average Age **(years)**	N=50 50.49 ±11.48	N=35 35.62 ±13.76	N=20 51.15 ±11.37
Age Groups			
< 45 **years** 46-54 **years** > 55 **years**	20 (40%) 16 (32%) 14 (28.7%)	14 (70.0%) 2 (10%) 4 (20%)	12 (45%) 13 (37%) 10 (28%)
Tumor size			
**0-2 cm** **>2 cm**	23 (46%) 27 (54%)		
Grade			
**1** **2** **3**	8 (8.9%) 28 (62.2%) 13 (28.9%)		
Stage			
**1** **2** **3**	16 (32 %) 19 (38%) 15 (30%)		
ER / PR			
**ER +/PR +** **ER -/PR -**	31 (62.6%) 19 (38%)		
Her2			
**Positive** **Negative**	19 (38%) 31 (62%)		
ER / PR / Her2			
**Non-TNBC** **TNBC**	36 (72%) 14 (28%)		
Ki67			
**<16%** **16-40%** **>40%**	24 (48%) v 15 (30.%) 11 (22.%)		

ER: Esterogen Receptor; PR: Progesterone Receptor; HER2: Human Epidermal growth factor Receptor 2

**Table 2:** RT primers used for cDNA synthesis (stem-loop method)

miRBase accesion code.	Gene name	RT stem-loop
MIMAT0005886	hsa-miR-1297	5’ GTCGTATCCAGTGCAGGGTCCGAGGTATTCGCACTGGATACGACCACCTGA 3’
MIMAT0018951	hsa-miR-4435	5’ GTCGTATCCAGTGCAGGGTCCGAGGTATTCGCACTGGATACGACCCTCTGTG 3’
MIMAT0022732	hsa-miR-3191-5p	5’ GTCGTATCCAGTGCGTGTCGTGGAGTCGGCAATTGCACTGGATACGACTGGAAG 3’
MIMAT0018992	hsa-miR-4465	5’ GTCGTATCCAGTGCAGGGTCCGAGGTATTCGCACTGGATACGACTCCCCTG 3’
MIMAT0000069	hsa-miR-16-5p	5’ GTCGTATCCAGTGCAGGGTCCGAGGTATTCGCACTGGATACGACCGCCAAT 3’
>NR_004394.1	U6 snRNA RT	5’AAAATATGGAACGCTTCACGAATTTG3’

miR: microRNA; RT: reverse transcription

**Table 3:** Primer used for qPCR

Gene name	Forward and reverse primers
hsa-miR-1297	F: 5’ CGGCGGTTCAAGTAATTCAGGTG 3’ R: 5’ CCAGTGCAGGGTCCGAGGTA 3’
hsa-miR-4435	F: 5’- CCAGAGCTCACACAGAGGG 3’ R: 5’ TCGTATCCAGTGCAGGGTCC 3’
hsa-miR-3191-5p	F: 5’ GGCTCTCTGGCCGTCTACC 3’ R: 5’ CCAGTGCAGGGTCCGAGGTA 3’
hsa-miR-4465	F: 5’ CCGCTCAAGTAGTCTGACCA 3’ R: 5’ AGTGCAGGGTCCGAGGT 3’
hsa-miR-16-5p	F: 5’ GGTAGCAGCACGTAAATATTGGC 3’ R: 5’ TCGTATCCAGTGCAGGGTCC 3’
U6 snRNA	F: 5’ TCGCTTCGGCAGCACATATAC 3’ R: 5’ CTTCACGAATTTGCGTGTCATCC 3’

**Table 4:** Separation of normal and malignant samples using logit(p)= 0.265 as cutoff value

parameter	Normal	Malignant	Total
Test positive*	2	21	23
Test negative**	17	3	20
Total	19	24	43
*logit(p)>0.265, ** logit(p)<=0.265			

## Discussion

Various studies have shown the expression of miRs in serum and tumors of patients with breast cancer (39-42). Our findings are in conformity with a number of recent studies, showing that the mean expression of miR-1297 was down-regulated in a number of malignant tumors, including gastric, colorectal, pancreatic, and lung cancer compared with matched adjacent non-tumor tissues (30, 32). However, some studies have shown that the mean expression levels of miR-1297 were up-regulated in the malignant breast tissues, testicular germ cell tumors, and laryngeal squamous cell carcinoma (19, 20). Up-regulation of miR-1297 expression was also shown to be significantly correlated with advanced stage, TNM, and larger tumor size (43). However, these clinical analyses are not supported by our findings. These inconsistencies might be explained by adoption of different experimental approaches.

No data have been reported on the expression of miR-4465, miR-3191-5p, and miR-4435 in breast cancer patients. In a study, down-regulation of miR-3191-5p expression has been shown in patients with hepatitis B virus (HBV) infection compared with that of controls (44). 

We found that miR-4465 expression level in malignant breast tumors was significantly amplified when compared with that of matched, adjacent non-tumor tissues. On the contrary, reduction in miR-4465 expression has been shown in NSCLC. Moreover, we found that the expression of miR-3191-5p was correlated with the size and ki-67 of tumor score, which may suggest it as an onco-miR for cell proliferation. However, an inverse correlation was shown for miR-4465 with tumor grade and tumor ki-67% score, which may suggest it as a possible tumor suppressor miR. In a study, up-regulation of miR-4465 expression has been reported in panic disorder (31), which may explain why we found such a result, since cancer causes panic in patients.

Association of circulating other miRs with tumor ER, PR, and Her2 status have been described in other studies (21, 34, 40, 45). Interestingly, this relationship was also shown in the present study since higher expression level of miR-3191-5p and lower expression level of miR-1297 were correlated with HER2 negative status. 

The diagnostic efficacy of combining three and four miRs (miR-148b, miR-376c, miR-409-3p and miR-801, miR-148b, miR-409-3p and miR-801) were evaluated. AUCs for miRs were 0.64 to 0.66, while AUC for three-marker combination was 0.69 (46). For breast cancer detection, a panel of nine miRs (miR-15a, miR-18a, miR-107, miR-133a, miR-139-5p, miR-143, miR-145, miR-365, and miR-425) has been presented with a corresponding AUC=0.665 (21). In triple negative breast cancers, a 4-miRNA signature given by miR-30e, miR-27a , miR-155 and miR-493 expression levels has been suggested as a diagnostic biomarker with a sensitivity 0.75 and a specificity 0.56; AUC=0.74 (33). In the present study, to discriminate malignant from normal and from benign subjects, AUCs of 0.700 to 0.97 and of 0.789 to 0.981 were obtained according to ROC curve analysis, miR-3191-5p has discriminated breast cancer from control subjects, yielding an AUC of 0.976 with a sensitivity of 86.67% and a specificity of 92%. Moreover, for the differentiation of malignant from benign tumors, AUC of 0.981 with a sensitivity of 93.33 % and a specificity of 94.12%, is suggested.

## Conclusion

This study revealed that to discriminate malignant from control and benign status, combination of four-miRs, as a possible biomarker, can be recommended. Therefore, these miRs are suggested as possible onco- (miR-4465) or tumor suppressor (miR-3191-5P, miR-1297, miR-4435) miRs. However, due to the small number of subjects studied in the present study, a larger-scale investigation is required to validate these data.
